# Comparing a Fitbit Wearable to an Electrocardiogram Gold Standard as a Measure of Heart Rate Under Psychological Stress: A Validation Study

**DOI:** 10.2196/37885

**Published:** 2022-12-21

**Authors:** Joel Gagnon, Michelle Khau, Léandre Lavoie-Hudon, François Vachon, Vicky Drapeau, Sébastien Tremblay

**Affiliations:** 1 School of Psychology, Faculty of Social Sciences Laval University Québec, QC Canada; 2 Faculty of Social Sciences Laval University Québec, QC Canada; 3 Quebec Heart and Lung Institute Research Center, Department of Physical Education, Faculty of Educational Sciences Centre Nutrition, santé et société (NUTRISS), Institute of Nutrition and Functional Foods (INAF) Laval University Québec, QC Canada

**Keywords:** Fitbit device, wearable, heart rate, measurement accuracy, criterion validity, interdevice agreement, psychological stress, stress, physiological, behavioral, mental health, well-being

## Abstract

**Background:**

Wearable devices collect physiological and behavioral data that have the potential to identify individuals at risk of declining mental health and well-being. Past research has mainly focused on assessing the accuracy and the agreement of heart rate (HR) measurement of wearables under different physical exercise conditions. However, the capacity of wearables to sense physiological changes, assessed by increasing HR, caused by a stressful event has not been thoroughly studied.

**Objective:**

This study followed 3 objectives: (1) to test the ability of a wearable device (Fitbit Versa 2) to sense an increase in HR upon induction of psychological stress in the laboratory; (2) to assess the accuracy of the wearable device to capture short-term HR variations caused by psychological stress compared to a gold-standard electrocardiogram (ECG) measure (Biopac); and (3) to quantify the degree of agreement between the wearable device and the gold-standard ECG measure across different experimental conditions.

**Methods:**

Participants underwent the Trier Social Stress Test protocol, which consists of an oral phase, an arithmetic stress phase, an anticipation phase, and 2 relaxation phases (at the beginning and the end). During the stress protocol, the participants wore a Fitbit Versa 2 and were also connected to a Biopac. A mixed-effect modeling approach was used (1) to assess the effect of experimental conditions on HR, (2) to estimate several metrics of accuracy, and (3) to assess the agreement: the Bland-Altman limits of agreement (LoA), the concordance correlation coefficient, the coverage probability, the total deviation index, and the coefficient of an individual agreement. Mean absolute error and mean absolute percent error were calculated as accuracy indices.

**Results:**

A total of 34 university students were recruited for this study (64% of participants were female with a mean age of 26.8 years, SD 8.3). Overall, the results showed significant HR variations across experimental phases. Post hoc tests revealed significant pairwise differences for all phases. Accuracy analyses revealed acceptable accuracy according to the analyzed metrics of accuracy for the Fitbit Versa 2 to capture the short-term variations in psychological stress levels. However, poor indices of agreement between the Fitbit Versa 2 and the Biopac were found.

**Conclusions:**

Overall, the results support the use of the Fitbit Versa 2 to capture short-term stress variations. The Fitbit device showed acceptable levels of accuracy but poor agreement with an ECG gold standard. Greater inaccuracy and smaller agreement were found for stressful experimental conditions that induced a higher HR. Fitbit devices can be used in research to measure HR variations caused by stress, although they cannot replace an ECG instrument when precision is of utmost importance.

## Introduction

The health and well-being of the population are a growing concern for clinicians and researchers. The World Health Organization (WHO) reported that noncommunicable diseases (eg, heart disease, stroke, cancer, diabetes, and chronic lung disease) were the cause of 71% of deaths worldwide in 2017 [1]. In addition, the exceptional regulatory measures taken to fight the COVID-19 pandemic have had a negative impact on the psychological health of citizens as made evident by a surge in incidences of psychological crises [2]. The extraordinary context generated by the COVID-19 pandemic has posed unprecedented challenges for governments [3,4] and has led to a redefinition of human activities within our societies, namely an acceleration in the digital transformation of health care [5]. The limitations of the current human health management model, which operates primarily downstream (ie, once problems emerge), are increasingly apparent. Moreover, the prevalence of health problems in the world far exceeds the current capacity of professionals and health services [6]. With the ever-increasing presence of technology in human life, researchers have taken an interest in the use of consumer wearable devices, such as smartwatches, armbands, rings, and other accessories, designed to be worn all day, to collect real-time physiological (eg, cardiac activity, skin temperature) and behavioral measures (eg, frequency of physical activity, step count, and sleep patterns). Moreover, these measures can be used to identify individuals at risk of declining mental health and well-being. Importantly, this information may provide relevant insight into the early detection and prevention of disease and well-being deterioration [7,8].

Regarding the validity and reliability of wearable activity monitors, systematic reviews have shown that these devices are somewhat accurate and stable to estimate heart rate (HR) and step count in adults. However, they provide an unreliable estimate for energy expenditure under different activities [9-12]. First, as several studies have pointed out, the sensors used to detect HR in most wearables (including Fitbit) are more sensitive to motion-induced artifacts (signal interference) than electrocardiogram (ECG) technology [13-15]. Accordingly, insufficient pressure and sensor-skin contact, as well as too much pressure such that blood flow was constricted, can affect HR measures. Despite these shortcomings, wearable activity monitors can provide important insight into physiological patterns. Importantly, small longitudinal studies have found support for the use of wearables to measure stress levels among adults [16,17]. Sano et al [18] conducted a monthlong longitudinal study of 201 university students to evaluate the possibility to predict mental health and stress using data collected with Q-sensor and Motion Logger wearable devices. The results revealed electrodermal activity (ie, skin conductance and temperature) as a predictor of mental health and stress. Additionally, several studies have revealed the variability in HR to be a valid indicator of stress and have applied this measure in the study of major depressive disorder, stress resilience, stress regulation, and recovery from mental and physical stress [19-22], although to our knowledge, only one study has examined the feasibility of using wearable activity monitors to measure HR as a direct indicator of stress [23]. However, a limitation of this study was the lack of comparison measures. As such, the validity of the relationship between wearable activity monitors measured HR and stress, as well as the potential applications, remain unclear. Moreover, to our knowledge, no laboratory study has assessed the relationship between HR and psychological stress using a wearable activity monitor. Thus, the aim of this study was threefold: (1) to test the ability of a wearable activity monitor, specifically a Fitbit device, to sense an increase in HR upon induction of psychological stress; (2) to assess the accuracy (ie, the closeness of the agreement between the result of a measurement and a true value of the thing being measured [24]) of a Fitbit device to capture a physiological change (increased HR) caused by psychological stress compared to a gold-standard ECG measure; and (3) to quantify the degree of agreement (ie, the degree of concordance or extent to which one measure can replace another) between the Fitbit device and a gold-standard ECG measure across different experimental conditions.

## Methods

### Recruitment

Participants were recruited through the mailing list from the authors’ university. Eligibility criteria were being between 18 and 65 years of age; being registered as a full-time student; having access to a smartphone; absence of current or past, non-BMI-related, pathology (somatic, psychiatric, or both); not taking painkillers, medications that affect the heart rhythm, or medication for major depression or other mood disorders; not being pregnant or breastfeeding; and understanding French (spoken and written).

### Ethical Considerations

This study was approved by the Human Research Ethics Committee of Université Laval (2020-053/10-11-2020). French-speaking participants provided written informed consent in French prior to participation in the study. All study data were deidentified to protect the privacy and confidentiality of participants. Upon completion of the study, the participants received a CAD $40 (US $29.31) monetary compensation.

### Participants

A total of 34 healthy university students were recruited for this study. Participant demographic data are presented in Table 1. The study occurred during the winter 2021 and summer 2021 semesters.

**Table 1:** Participant demographic data (N=34).

Demographics		Study sample
Age (years), mean (SD)		26.8 (8.5)
**Gender, n (%)**		
	Male	13 (35)
	Female	21 (58)
	N/A^a^	2 (5)
**Ethnicity, n (%)**		
	Caucasian	23 (67)
	African	4 (11)
	Hispanic	3 (8)
	N/A	1 (2)
	Middle Eastern	2 (5)
**Education level, n (%)**		
	Bachelor	14 (41)
	Master	10 (26)
	Doctoral	8 (23)
	N/A	3 (8)
**Program of study, n (%)**		
	Health Science	8 (23)
	Science and Engineering	5 (14)
	Languages	3 (8)
	Arts and Humanities	3 (8)
	Psychology	3 (8)
	Social Science	2 (5)
	Administration	2 (5)
	Education	1 (2)
	N/A	7 (20)

^a^N/A: not applicable.

### Fitbit Device as an HR Monitor

Fitbit (Fitbit Inc) is one of the most popular wearable activity monitors and the most frequently studied [9]. While Fitbit’s market shares have diminished from its peak in 2018, as of 2019, the company remains in the top 5 wearable companies by shipment volume and market share [25]. Recent studies have investigated the capacity of Fitbit devices to measure HR under different exercise conditions. Benedetto et al [26] in their controlled assessment of the Fitbit Charge 2 accuracy in measuring HR found wide variability in precision during different intensities on a stationary bike. In another study, Thomson et al [27] compared the HR measurement of the Apple Watch and the Fitbit Charge HR 2 with an electrocardiogram (ECG) among healthy young adults across different treadmill exercise intensities. The results showed diminished accuracy with increased exercise intensities for all devices, while the Fitbit had comparably greater relative error rates (ranging from 4.91% for very light exercise to 13.04% for very vigorous exercise) compared to the Apple Watch. Indeed, a general lack of accuracy at higher exercise intensities has been repeatedly reported in the literature for Fitbit devices [28-30]. Nevertheless, Fitbit data may still be useful for other purposes, such as providing a proxy measure of psychological stress, as investigated within the context of the present study.

### Experimental Procedure

Students interested in the study were invited to the laboratory. Upon arrival, participants were asked to read and sign an informed consent document. Participants who consented to the study were then asked to complete a web-based self-reported questionnaire to gather sociodemographic information. Next, they were asked to install the wearable activity monitor’s (Fitbit) mobile app on their cell phone. In order to assess Fitbit detection of stress-induced change in HR, the participants were then given a Fitbit Versa 2 to wear on their nondominant hand during the experiment. Participants were asked to sit in front of 3 cameras. Once seated, the research assistant installed 3 electrodes, located on the left and right of the chest, as well as below the ribs on the left of the abdomen of the participant. Once the electrodes were placed, the research assistant started the physiological recordings and made a visual inspection of the signal to ensure that the electrodes made good contact with the skin. Afterward, the research assistant started the stress protocol (described below). At the end of the stress protocol, the research assistant debriefed the participant and explained the purpose of the protocol. This step was important to ensure that the participant would not experience anger toward the research assistant when leaving. The experimental procedure took on average 1 hour and 30 minutes to complete.

#### Stress Protocol

The Trier Social Stress Test (TSST) was used to induce psychological stress. The TSST is a standardized psychosocial stress test that has been extensively used by researchers worldwide [31]. The TSST has been recognized to be an especially successful way of triggering stress [32]. The TSST consists of a waiting period, stress period, and rest period which can be divided into 5 experimental phases: relaxation, anticipation, oral, arithmetic, and relaxation. During the waiting period (the relaxation phase), the participant was left alone in the room and was told to relax for 5 minutes. The stress period was divided into 3 parts. First, a 3-minute anticipatory stress period (anticipation phase) during which the participant was asked to prepare a speech about why they would be a good candidate for their dream job. Second, a 5-minute speech task (oral phase) during which they delivered their speech in front of the research assistant. The research assistant was instructed to prompt the participant to continuously talk for the entire 5 minutes. If the participant were to stop talking before the end of the condition, the research assistant would use verbal prompts to pressure the participant to continue talking. Third, immediately after the speech task, the participant was asked to verbally perform a 5-minute mental arithmetic task (arithmetic phase). For this task, the participant was required to continuously subtract 13 from the number 1687. If the participant made a mistake or hesitated for more than 3 seconds, the research assistant triggered a loud buzzer and instructed the participant to start again from the initial number. Following the arithmetic task, the participant was asked to relax for 5 minutes (the relaxation phase). During the TSST, the participant was filmed from 3 angles (front, 45° left, and 45° right) and was informed that these recordings would be analyzed by 2 language analysis experts.

### Material and Measures

#### Fitbit Versa 2

The experimental device was the wrist-worn Fitbit Versa 2, Version 35.72.1.9 (Fitbit Inc). Fitbit HR data were retrieved from the Fitabase platform (Small Steps Labs) and then stored in a secure S3 bucket maintained by the authors’ university for analysis.

#### Electrocardiography

Surface electrodes were used for ECG recordings using a Biopac MP150 acquisition system for physiological data acquisition. The electrodes recorded electrical impulses from the heart. Data were sampled at 1000 Hz. The recording was performed by a NeuroScan system (NeuroScan Inc, SynAmps). Heartbeats per minute (bpm) were calculated as an indicator of physiological stress arousal.

### Statistical Analysis

Descriptive statistics for HR measurements (mean and standard deviation) were calculated for the Fitbit Versa 2 and the Biopac across experimental phases (relaxation, anticipation, oral, and arithmetic). The relaxation phase at the beginning and the one at the end of the experimental protocol were merged to create a single relaxation phase for the analyses.

First, to assess the ability of the Fitbit Versa 2 to sense an increase in HR, differences across experimental phases were examined using a mixed-effect model that was constructed using the “lme4 [33]” package available in R (R Foundation for Statistical Computing). The model included the experimental phases as a fixed effect, whereas the participants and the interaction between participants and experimental phases were treated as random effects. For the overall model, a Satterthwaite adjustment was used to compute the degrees of freedom. Partial η^2^ was computed using the “effectsize [34]” package available in R. Post hoc tests were conducted using the “emmeans [35]” package available in R with the Kenward-Roger method to compute the degrees of freedom, and the *P*-values were adjusted using the Tukey method.

Second, to assess the accuracy of the Fitbit Versa 2 measured HR compared to a gold-standard ECG measure, mean absolute error (MAE), and mean absolute percentage error (MAPE) between the Biopac and the Fitbit Versa 2 were calculated as overall measurement error. The clinically acceptable difference (CAD) was set as 10 bpm, such that differences in MAE of less than 10 bpm were regarded as clinically insignificant, thus showing good accuracy for the Fitbit Versa 2. This was based on the American National Standard of “Cardiac monitors, heart rate meters, and alarms” that permit “readout error of no greater than ±10% of the input rate or ±5 bpm, whichever is greater [36].” A MAPE threshold of 10% was used to assess the accuracy of the Fitbit Versa 2 [37,38].

Third, to quantify the degree of agreement between the Fitbit Versa 2 and the Biopac across different experimental phases, 5 metrics of agreement were calculated. First, limits of agreement between the Biopac and the Fitbit Versa 2 were evaluated using a mixed-effect model to account for the effect of the participant, the experimental phases, and time (ie, repeated measures) as recommended by Parker et al [39]. Bias-corrected and accelerated bootstrapping with 5000 replications were used to estimate the 95% CI. The analysis was conducted using the “SimplyAgree [40]” package in R. Second, the concordance correlation coefficient (CCC) with 95% CI was estimated from a linear mixed model using the appropriate intraclass correlation coefficient [41]. The CCC indicates the proportion of the total variability accounted for (1) by the participant, (2) the experimental phase, and (3) their interaction. The CCC is a standardized coefficient taking values from 1 (perfect disagreement) to 1 (perfect agreement). In other words, a CCC of 1 indicates the absence of variability in the device across participant and experimental phases [42]. In this study, the following guidelines [43] were used to interpret the CCC: <0.90 (poor), 0.90 to 0.95 (moderate), 0.95 to 0.99 (substantial), and >0.99 (perfect). The CCC was calculated using the “cccrm” package in R [44]. Third, the coverage probability index proposed by Lin et al [45] was estimated by calculating the probability that the between-device differences lie within the boundary of the predefined CAD. As such, a larger probability indicates closer agreement. A mixed-effect modeling approach was used to calculate the coverage probability index, which required the range of CADs and the mean square deviation. The mean squared deviation is the expected squared difference between readings by 2 different devices on the same individual performing the same activity at the same time. Similar to Parker et al [42], the mean squared deviation was obtained based on the mixed-effect model. Fourth, the total deviation index was estimated based on the mean squared deviation from the mixed-effect model. This index provides the boundary within which the differences between devices will be contained p × 100% of the time. The predefined CAD of ±10 bpm was used to interpret whether the interval signified agreement. An interval contained between the CAD would indicate that the 2 devices can be used interchangeably. Finally, the coefficient of individual agreement (CIA [46,47]) was calculated. The CIA is a scaled coefficient that quantifies the magnitude of variability between devices compared to the replication variability within devices. A CIA value of 1 indicates that using different devices makes no difference to the variability of repeated measurements taken under the same conditions within the same subject. Following past studies [42,48,49], the CIA was calculated based on the mean squared deviation as the disagreement index. A CIA >0.80 is considered acceptable [46,48,49]. A 95% CI was calculated using a bootstrapping procedure with 5000 replications. All statistical analyses were conducted using R version 4.0.3 [50].

## Results

### HR Mean Differences Across Experimental Phases

**Figure 1:**
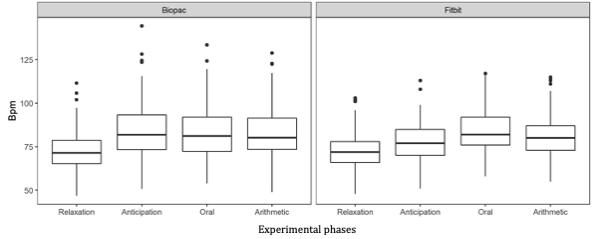
Boxplots of the bpm across experimental phases and between devices. bpm: beats per minute.

Figure 1 displays boxplots of the bpm across experimental phases and between devices. The results from the mixed-effect model revealed significant variations from the Fitbit Versa 2 HR measurements among experimental phases (*F*_3,103_=44.03; *P*<.001; η^2^_partial_=0.56, 90% CI 0.45-0.64). A post hoc Tukey test revealed that the mean HR from the relaxation phase was significantly lower than all other experimental phases (anticipation, oral, and arithmetic) at *P*<.001. Furthermore, the mean HR from the anticipation phase was significantly lower than the oral (*P*<.001) and arithmetic (*P*=.02) phases. Finally, the mean HR from the oral phase was significantly higher than the arithmetic phase (*P*=.02). Overall, the results revealed the capacity of the Fitbit Versa 2 to detect short-term variations in levels of psychological stress.

### Accuracy

Analysis of the measurement error between the Biopac and the Fitbit Versa 2 showed an overall MAE of 5.87 (SD 6.57, 95% CI 3.57-8.16) bpm, which is below the predefined CAD of ±10 bpm showing good accuracy of the Fitbit Versa 2. Moreover, the results revealed an overall MAPE of 7.24% below the predefined threshold of 10% for acceptable accuracy. Table 2 shows the MAE (and SD) and MAPE for each experimental phase.

**Table 2:** Accuracy of the Fitbit Versa 2 across experimental phases.

Phase	Mean absolute error (SD)	95% CI	Mean absolute percentage error (%)
Relaxation	4.32 (4.92)	2.03-6.61	6.08
Anticipation	8.94 (8.92)	6.65-11.24	9.83
Oral	6.60 (6.33)	4.30-8.89	7.88
Arithmetic	6.13 (6.88)	3.84-8.43	7.16

### Agreement

**Figure 2:**
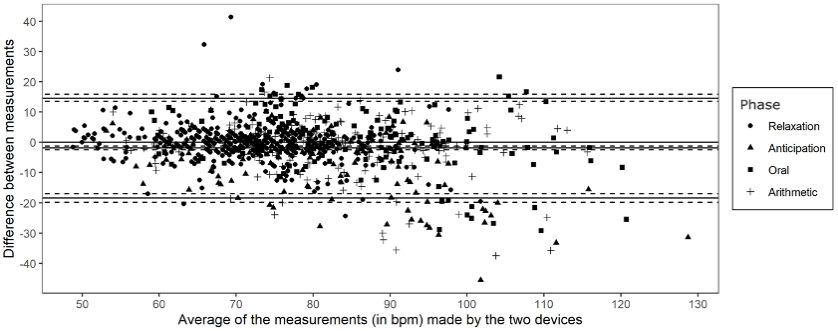
Bland-Altman plot. Mean bias and limits of agreement are shown by the full lines, while confidence intervals are shown by the dashed lines. bpm: beats per minute.

Results from the mixed-effect limits of agreement method revealed a mean bias of −1.91 (95% LoA −18.37 to 14.58). Figure 2 shows the corresponding Bland-Altman plot with its 95% LoA. The CCC was estimated to be 0.76 (95% CI 0.66-0.83). The coverage probability index with a CAD of ±10 bpm was estimated to be 0.72 (95% CI 0.66-0.81). The mixed-effect model estimated a mean squared deviation of 85.69 (95% CI 57.84-111.51). The total deviation index was calculated to be 18.14 (95% CI 14.91-20.70). Prior to analyzing the CIA, the residual error variance was calculated. The results showed a Bland-Altman repeatability coefficient of 15.11, which signifies an approximate 95% probability that the repeated bpm values are within 15 bpm of each other. The CIA was estimated to be 0.69 (95% CI 0.57-0.84).

The variance component estimates of the mixed-effect model were evaluated to find the principal sources of disagreement (Table 3). Results showed substantial within-subject variability (*σ*^2^=116.57). Moreover, the variability of the experimental phases (*σ*^2^=22.58), within-subject residual (*σ*^2^=29.72), and the subject-phase interaction (*σ*^2^*=*14.47) was high. To better understand the effect of the specific experimental phase on the agreement between devices, mixed-effect models were analyzed for each phase separately.

In sum, results revealed that, when compared with a gold-standard device, the Fitbit Versa 2 shows overall poor agreement on all metrics analyzed. Further analyses conducted for each experimental phase revealed adequate agreement during the relaxation phase, whereas the preparation phase showed the worst agreement between the 2 devices (Table 3).

**Table 3:** Metrics of agreement between the Fitbit Versa 2 and the gold-standard ECG across experimental phases.

Phase	LoA^a^ (95% LoA)	Concordance correlation coefficient (95% CI)	Coverage probability index (95% CI)	Total deviation index (95% CI)	Coefficient of individual agreement (95% CI)
Relaxation	−0.27 (−8.70 to 8.16)	0.78 (0.67 to 0.84)	0.85 (0.79 to 0.90)	13.76 (11.85 to 15.69)	0.98 (0.88 to 0.99)
Preparation	−6.70 (−20.36 to 6.97)	0.56 (0.44 to 0.67)	0.55 (0.49 to 0.67)	25.94 (20.18 to 29.93)	0.52 (0.35 to 0.69)
Oral	0.65 (−11.08 to 12.38)	0.81 (0.71 to 0.87)	0.74 (0.68 to 0.83)	17.37 (14.17 to 19.74)	0.61 (0.41 to 0.79)
Arithmetic	−1.32 (−12.95 to 10.31)	0.74 (0.58 to 0.87)	0.71 (0.62 to 0.88)	18.42 (12.70 to 22.23)	0.59 (0.38 to 0.86)

^a^LoA: limits of agreement.

## Discussion

### Main Study Findings

Regarding the first objective of testing the ability of a Fitbit device to sense an increase in HR upon induction of psychological stress, results from a mixed-effect model revealed that the HR measurements from the Fitbit Versa 2 showed significant mean differences across all experimental phases. Regarding the second objective of assessing the accuracy of a Fitbit device to capture change in HR compared to a gold-standard ECG, the MAE index and MAPE results showed acceptable accuracy across all phases. Regarding the third objective of quantifying the degree of agreement between the Fitbit device and the gold-standard ECG measure, the 95% CI was fairly large and lies outside of the predefined CAD, indicating that the 2 devices did not reach the desired LoA. Moreover, the 95% CI indicated that HR measurements could be underestimated by almost 19 bpm. Visual inspection of the Bland-Altman plot showed small differences between the 2 devices for bpm <90 and greater differences for higher bpm.

Unsurprisingly, the differences in HR detected across experimental phases were markedly more important between the relaxation condition and the oral and arithmetic conditions, a pattern that is echoed in previous studies that have investigated HRV using the TSST [51-53]. These results are also in line with longitudinal research that found support for the use of wearable devices to measure stress levels [17-19,26] and indicate that short-term variations in levels of psychological stress can be detected using a Fitbit device. Additionally, although this is the first study to quantify the accuracy of a Fitbit device under experimentally induced psychological stress, accuracy estimates from Fitbit devices under different exercise intensities have been published [27-29]. Regarding the overall MAE, results from this study revealed similar accuracy to what has been found in a past study comparing a Fitbit device to an ECG gold standard [25]. In this study, MAPE estimates show a loss in accuracy under the stressful phases compared with the relaxation phase. Similar patterns were found in past studies where lower MAPEs were associated with light exercise and higher MAPEs with more vigorous exercise [25,28]. Overall, the evidence from several studies including the present one showed that under a normal or relaxed state, Fitbit devices provide accurate HR measurements. However, a loss in the accuracy of these devices can be observed, especially under high HR-inducing physical or psychological stress. Finally, though Bland-Altman LoA revealed a small mean bias compared to previous studies on exercise intensities [25], results regarding the degree of agreement between both devices echo previous findings revealing that wearable devices tend to not perform well compared to gold-standard devices at higher bpm conditions [25,26,54]. Interestingly, the highest mean bias was found in the anticipation phase. The high variability in HR measurements across participants from both devices (especially from the Biopac) may partly explain this result. This variability may emerge from individual differences, in coping with anticipation of a stressful event, especially since the majority of participants were female. Indeed, previous research indicates that men tend to show higher levels of stress than women during the anticipation of a psychosocial stress task [55].

The CCC indices found in this study consistently showed poor agreement between the devices. When compared to past research on physical activity, the CCC found in this study revealed better agreement than what has been found by Thomson et al and worse agreement than the results from Wang et al [56]. Evaluation of the variance components of the mixed-effect model showed important between-subjects variability which is not surprising given the nature of the stressful phases used in this study. For example, some participants may experience more stress during a verbal task than others. A review found that 30% to 50% of people have a fear of public speaking with 40% reporting anxiety about being negatively evaluated by others [57]. Moreover, interparticipant variability in HR changes is echoed in a previous study that used Fitbit-measured HR as an indicator of stress [24]. As such, to account for this expected variability, the CIA was computed as it is less dependent on the between-subjects variability compared to the CCC [47,58]. However, the repeatability coefficient of Bland-Altman was found to be unacceptably high and warrants caution when interpreting the CIA. Based on past guidelines suggesting a value of at least 0.80 to conclude good agreement [46,49], the overall estimated CIA in this study suggested poor agreement between the devices. The only CIA that reached a good agreement was for the relaxation phase indicating the similarities between the two devices for low bpm. This result provides further evidence that Fitbit devices tend to show greater precision for low bpm conditions for physical activities [25,26].

The estimated overall coverage probability index (CPI) was well below the predefined 0.95 threshold to represent reasonable agreement, suggesting unsatisfactory agreement between devices. Unsurprisingly, the lowest CPI estimate was found in the anticipation phase, which showed the largest between-subjects and between-devices variation in bpm. Results from the total deviation index (TDI) indicated that differences between the Biopac and the Fitbit Versa 2 are expected to lie within ±18.14 bpm 95% of the time. Compared with the predefined CAD of ±10 bpm, all TDI values showed poor agreement and were too large to conclude that the 2 devices could be used interchangeably. Overall, the indices of agreement computed in this study showed that the HR measurements from the Fitbit Versa 2 vary significantly from an ECG gold standard, especially for higher bpm. In light of these results, it appears that although the Fitbit Versa 2 can capture short-term variations in bpm under different stress and relaxation conditions, the precision of these variations is questionable.

#### Limitations

This study contains limitations that need to be acknowledged to fully appreciate its results. First, the sample size of 34 participants may be considered small and did not specifically exclude participants using substances of abuse that may affect their heart rate (eg, nicotine and alcohol). However, our recruitment criteria and size are comparable to previous similar studies that also did not specifically exclude individuals using substances of abuse and have sample sizes that range from 15 to 50 participants [26-29]. Second, the levels of psychological stress were experimentally induced in a controlled laboratory setting, and further research is needed to test whether these results also apply in natural living conditions. However, the psychological stress and relaxation conditions were induced using a well-validated protocol (TSST). Moreover, efforts were made to ensure rigor in analyses, namely through the use of 5 different metrics estimated with their corresponding 95% CI to determine agreement between the Fitbit Versa 2 and the gold-standard device. Third, we used the Biopac as the gold-standard device for measuring HR. While this ECG-based instrument provides medical-grade HR data, it involves the use of electrodes which, when placed incorrectly, can generate noise in the signal and even lead to less accurate data [59]. Despite this potential limitation, the authors believe it was important to have a gold-standard device with which to compare the Fitbit device for the purpose of concurrent validation. Fourth, we did not consider the skin color or the skin photosensitivity, factors that have previously been suggested to affect the signal resolution of sensors that use photoplethysmography technology such as Fitbit. In this study, over 30% of the participants were non-Caucasian, this could have also affected the accuracy of Fitbit readings [60,61]. Similarly, individual variables of body mass index, prior level of physical activity, and presence of symptoms of psychological disorders (with or without a diagnosis) were beyond the scope of this research and therefore not considered during analyses. However, these variables should be considered in future research interested in quantifying the impacts of individual variables on HR measurements. Finally, while the Fitbit Versa 2 was found to be able to capture short-term stress variation, longitudinal studies are needed before concluding on the potential of this device to capture mid-to-long-term stress levels to predict psychological distress and diminished well-being. Nevertheless, a strength remains that this is the first study to quantify the accuracy of a Fitbit device under experimentally induced psychological stress and can serve as an important foundation for future research regarding wearable activity monitors and psychological stress.

#### Conclusions

With the ubiquity of wearable devices and the growing interest to use the data they provide in the health sector, research is needed to test the reliability and validity of these instruments. To our knowledge, this is the first study to test the accuracy and agreement of a wearable device (Fitbit Versa 2) under different psychological stress-inducing experimental conditions. Results showed that the short-term variations in psychological stress levels were successfully captured by the Fitbit Versa 2. Moreover, MAE and MAPE estimates were all below the predefined threshold of ±10 bpm, indicating acceptable accuracy of the Fitbit Versa 2. However, across the 5 metrics of agreement analyzed, results revealed poor agreement between the HR measurement from the Fitbit device and the Biopac. Importantly, the results of this study have implications in advancing research involving the use of wearable devices as it provides preliminary evidence that the HR measurement from the Fitbit Versa 2 can be used to detect psychological stress among a nonclinical adult population.

## Abbreviations

bpm: beats per minute
CAD: clinically acceptable difference
CCC: concordance correlation coefficient
CIA: coefficient of individual agreement
CP: coverage probability
ECG: electrocardiogram
HR: heart rate
LoA: limits of agreement
MAE: mean absolute error
MAPE: mean absolute percentage error
TDI: total deviation index
TSST: Trier Social Stress Test
WHO: World Health Organization

## Data Availability

The data sets and the R code generated and analyzed during this study are available at [62].
